# Performance of Manganese(III) Acetylacetonate in Solvent-Borne and High-Solid Alkyd Formulations

**DOI:** 10.3390/ma13030642

**Published:** 2020-02-01

**Authors:** Eliška Matušková, Jan Honzíček

**Affiliations:** Institute of Chemistry and Technology of Macromolecular Materials, Faculty of Chemical Technology, University of Pardubice, Studentská 573, 532 10 Pardubice, Czech Republic; st42098@student.upce.cz

**Keywords:** alkyd paints, manganese, primary drier, infrared spectroscopy, Raman spectroscopy, EPR spectroscopy

## Abstract

This paper reports a strong drying activity of manganese(III) acetylacetonate. It is documented on several solvent-borne and high-solid alkyd binders. Solubility problems, which often appear upon application of new primary driers, were overcome by use of dimethyl sulfoxide. Interestingly, intense coloration of the drier does not influence the transparent paint films due to in situ reduction to manganese(II) as evidenced by colorimetric measurements. Kinetics of the autoxidation process was investigated by infrared and Raman spectroscopy. For selected formulation, the effect of film thickness on through drying was estimated by infrared spectroscopy using attenuated total reflection sampling technique.

## 1. Introduction

Polyesters resins modified with fatty acids, commonly known as alkyd resins, are of increasing interest due to a high content of renewable feedstocks. Common glycerol-based resins of a high polymerization degree may reach up to 66% oil-length, which specifies content of the modifying plant oil. Binders based on four-functional pentaerythritol may contain up to 78% fatty acid building blocks when assumed an infinitive linear polyester chain. Even higher oil-lengths, ~85%, are commonly reached in oligomeric structures of so-called alkyd oils often used as high-solid binders [1,2,3]. In the case of alkyd resins, film formation consists of a solvent evaporation and chemical processes proceeding on unsaturated fatty acid chains. These chemical processes, known as autoxidation, start by a formal insertion of air-oxygen on activated C–H bond to produce hydroperoxides. These kinetically stable intermediates are decomposed by action of elevated temperature or additives known as primary driers to give alkoxyl and peroxyl radicals responsible for the formation of durable cross-linked structure [1].

Primary driers based on cobalt(II) carboxylates are currently widespread in paint-producing industry due to their strong catalytic activity and a low cost. Nevertheless, their extensive use could be restricted considerably in near future owing to their recently recognized carcinogenic properties [4]. This situation stimulates researchers and paint producers to search for new alternatives based on coordination compounds of less toxic metals such as manganese [5], iron [6,7,8], or vanadium [9,10,11,12].

**Scheme 1:**
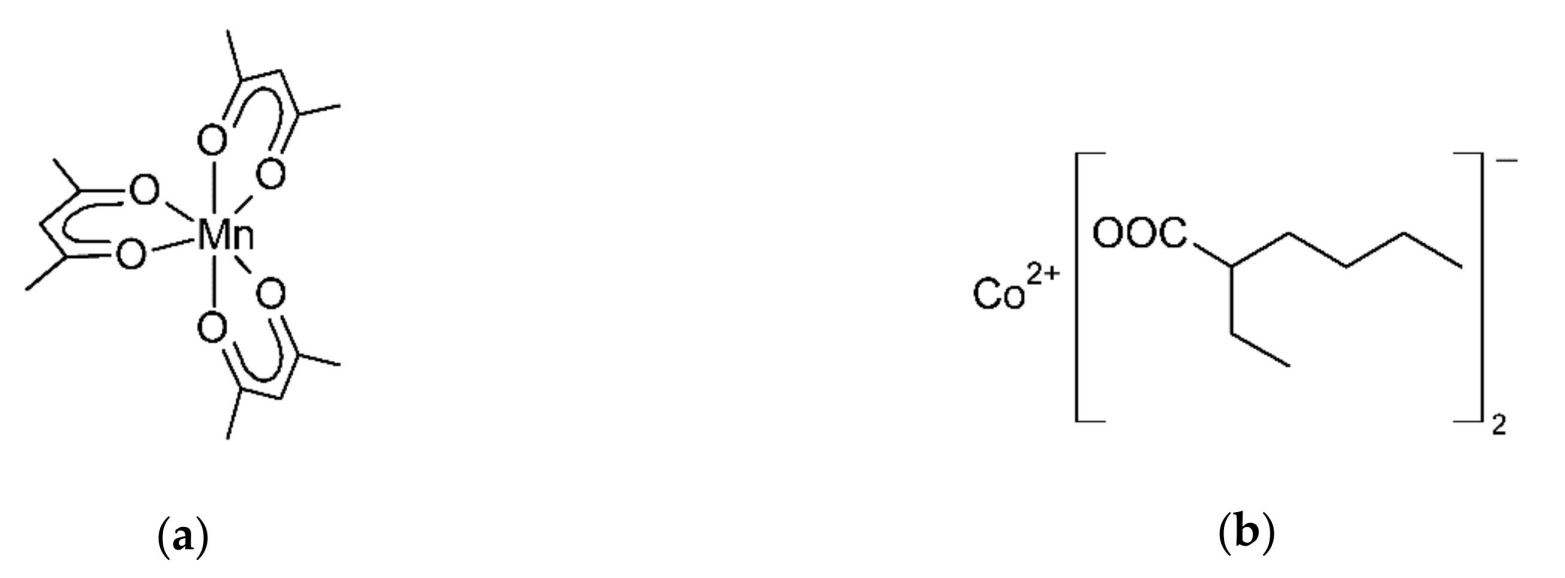
Chemical formulas of (**a**) manganese(III) acetylacetonate (Mn) and (**b**) cobalt(II) 2-ethylhexanoate (Co).

The field of manganese-based driers has been of particular interest for a long time and some of them are already commercially available, e.g., manganese(II) carboxylates and their mixtures with chelating ligands [1,13,14]. This work is focused on application of manganese(III) acetylacetonate (Mn, Scheme 1, Formula a). Such compound is known as initiator of radical polymerizations [15,16,17,18], mediator in organic synthesis [19,20] and catalyst of oxidation reactions [21,22]. Patent literature reports application of acetylacetone solution of Mn in alkyd-based paints, where it performs as the primary drier [23]. Such system exhibits a reduced skinning upon storage [23] probably due antioxidant properties of the solvent [24]. The ability of Mn to catalyze autoxidation of ethyl linoleate, which is often used as a model system for alkyds, was studied by Ming and Bouwman [25,26]. They recognized improved activity of Mn, when used in a mixture with 2,2′-bipyridine that facilitates Mn(III) to Mn(II) reduction [26].

The aim of this contribution is to establish manganese(III) acetylacetonate (Mn) dissolved in dimethyl sulfoxide as a primary drier for solvent-borne and high-solid alkyd formulations and compare its performance with commercial cobalt(II) 2-ethylhexanoate (Co, Scheme 1, Formula b). The mechanic assays on test coatings will be supported by kinetic measurements of the autoxidation process performed on a thin layer of alkyd resin.

## 2. Materials and Methods

Manganese(III) acetylacetonate was prepared as described elsewhere [27]. The commercial cobalt-based driers cobalt(II) 2-ethylhexanoate (Co; 65 wt.% in mineral spirits) and Octa-Soligen Manganese 10 were obtained from Sigma-Aldrich (St. Louis, MO, USA) and Borchers (Langenfeld, Germany), respectively. Alkyd resins CHS-ALKYD S471 X 60 (**S471**; oil length 47%, acid value = 6 mg KOH/g), CHS-ALKYD S622 N 60 (**S622**; oil length 62%, acid value = 7 mg KOH/g), and CHS-ALKYD TI 870 (**TI870**; oil length 87%, acid value = 8 mg KOH/g) were supplied from Spolchemie (Ústí nad Labem, Czech Republic). NEBORES SP 262-50 (**SP262**; oil length 50%, acid value = 6 mg KOH/g), NEBORES SP 252-70 DMV (**SP252**; oil length 63%, acid value = 8 mg KOH/g), NEBORES FP 07-90 D (**FP07**; oil length 68%, acid value = 9 mg KOH/g) and NEBORES SP 00-100 (**SP00**; oil length 80%, acid value = 15 mg KOH/g) were supplied from Safic-Alcan (Brno, Czech Republic). Manganese(II) acetate tetrahydrate (Acros Organics, Geel, Belgium), dimethyl sulfoxide p.a. (DMSO; Lach-Ner, Neratovice , Czech Republic), toluene p.a. (Lach-Ner) and Thinner S 6006 Aroma free (Severochema, Liberec, Czech Republic) were used as obtained from supplier without further purification.

### 2.1. Preparation of Test Formulations

Alkyd formulations were prepared in the concentration range 0.1 to 0.01 wt.% of metal in dry matter content. Manganese(III) acetylacetonate was treated with 100 µl of DMSO. Immediately after dissolution, it was treated with 5 g of the alkyd resin. High-solid resins of dry matter higher than 90% were diluted with the thinner to 90%. The formulations were stirred vigorously with a spatula in glass vials and then degassed in the ultrasonic bath for 3 min in degas mode. The comparative formulation with the cobalt-based drier was prepared in similar way. The appropriate amount of commercial cobalt(II) 2-ethylhexanoate was diluted in 100 µl of toluene instead of DMSO.

### 2.2. Measurements of Drying Time

Prepared formulations of solvent-borne resins were applied on clean glass strips (305 × 25 × 2 mm^3^) using frame applicator of 76 µm gap. In case of high-solid alkyd resins, two sets of glass strips were coated with the formulations using frame applicators of 38 µm and 76 µm gaps. The drying performance has been determined using a B. K. Drying Time Recorder (BYK, Wesel, Germany) according to ASTM D5895-03 under standard laboratory conditions (T = 23 °C, relative humidity = 50%). The experiments were done in 24-h mode with hemispherical-ended needles (D = 1 mm) equipped with 5 g weights. Set-to-touch time (τ_1_), tack-free time (τ_2_), dry-hard time (τ_3_), and dry-through time (τ_4_) were estimated as follows; the film was found to be “set-to-touch” dry (τ_1_) when the wet film stopped flowing behind the needle. In the second stage, the needle gives a mark in the film revealing the glass substrate. After that, film was found to be “tack-free” dry (τ_2_). In the next stage, the needle starts to tear the film and leaves a dashed mark. This stage finishes when the film is “hard” dry (τ_3_). In the next stage, the needle leaves a continuous line on the film not tearing its surface. When no visible mark is observed, the film is “through” dry, i.e., “total” dry (τ_4_).

### 2.3. Determination of the Film Hardness

Film hardness was measured on test coatings applied on glass plates (200 × 100 × 4 mm^3^). The formulations were cast on the plates using frame applicators of 90 µm (high-solid binders) or 150 µm gaps (solvent-borne binders). Film hardness development was measured using a Persoz-Pendulum Damping Tester (Elcometer, Manchester, UK) according to ISO 1522:2006 under standard laboratory conditions (T = 23 °C, relative humidity = 50%) within 100 days. The method is based on registering the number of pendulum swings it takes before the amplitude of the pendulum is damped to a certain extent. Relative hardness (*H*_rel_) is related to number of swings obtained for a glass standard. The error in determination of surface hardness was estimated to ± 0.5%. 

### 2.4. Determination Film Coloration

Coloration of transparent films was measured on a UV–Vis Maya 2000 Pro spectrometer using halogen light source of DH-2000-BAL (Ocean Optics). The VIS spectra were processed in OceanView software (version 1.6.7, Ocean Optics, Dunedin, FL, USA) and expressed in CIELAB color space with a standard illuminant “D65” and an observer at “2-degrees”. The test formulations were cast on microscopic glass slides (76 × 26 × 1 mm^3^) using frame applicators of 120-µm gap and left under standard laboratory conditions (T = 23 °C, relative humidity = 50%) at diffuse daylight illumination. Reported data are given relative to pure microscopic slides.

### 2.5. Electron Paramagnetic Resonance (EPR)

Electron Paramagnetic Resonance (EPR) spectra were measured on Miniscope MS 300 spectrometer (Magnettech, Berlin, Germany) in microwave X-band (~9.5 GHz). The apparatus was gauged on DPPH value (*g* = 2.0036 ± 2). Solution samples were measured in glass capillaries (ID = 0.5 mm) at room temperature.

### 2.6. Infrared Spectroscopy

Kinetics of the drying process was followed by time-resolved infrared spectroscopy. The measurement was performed on a Nicolet iS50 FTIR spectrometer (Waltham, MA, USA) using attenuated total reflection (ATR) sampling technique (64 scans per spectrum, data spacing = 0.5 cm^−1^). The prepared test formulations were applied on a built-in diamond ATR crystal using a frame applicator of 25 µm gap. It gives coatings of 5 µm wet thickness as the crystal surface lies 20 µm above the plate, on which the frame applicator abuts. Thickness effect was evaluated in a similar way using frame applicators of 25, 45, 70, 120, 170, and 220 µm gaps. The mid-infrared spectra were recorded in region of 4000–400 cm^−1^ every 5 min for 24 h under laboratory conditions (T = 23°C; relative humidity = 50%). The collected spectra were integrated using a fixed two-point baseline in the region 3025–2990 cm^−1^ in order to estimate intensity of ν_a_(*cis*-C=*C–H*) band. Rate coefficients (*k*_max_) at the beginning of the autoxidation process were estimated as the steepest slope of the logarithmic plots of the integrated area vs. time. The intensity of ω(*cis-trans*-C=*C–H*) band was determined as height of the band at 989 cm^−1^ using a linear baseline fixed at the region 1010–945 cm^−1^.

### 2.7. Raman Spectroscopy

Raman spectra were recorded on a Nicolet iS50 spectrometer (Waltham, MA, USA) using FT-Raman module (Nd:YAG excitation laser λ = 1064 nm, power = 0.5 W, 256 scans per spectrum, data spacing = 1 cm^−1^) in region of 4000–200 cm^−1^. Test formulation was applied on a glass plate (200 × 100 × 4 mm^3^) using frame applicator of 60 µm gap to reduce effect of oxygen diffusion. Small samples of the coating were taken from the plate every 15 min and their spectra were immediately collected. Development of the spectra was followed for 5 h. Intensity of the collected spectra was calibrated on the intensity of ν_s_(*C–H*_arom_) band at 3074 cm^−1^. 

## 3. Results and Discussion

### 3.1. Performance in Solvent-Borne Alkyd Binders

The initial tests of drying activity was performed on four solvent-borne alkyd resins of medium (**S471** and **SP262**) and long oil length (**S622** and **S252**); all of them are commercial binders modified with semidrying soybean oil. Although manganese(III) acetylacetonate (Mn) is a dark-brown powder solid insoluble in organic solvents commonly used in paint-producing industry, the solubility problems were easily overcome by the use of dimethyl sulfoxide (DMSO) that was recently recognized as a promising candidate for replacement of volatile organic solvents in solvent-borne paints. It is a low volatile solvent classified as nontoxic with no risk for the human health [28]. 

The activity of Mn was investigated on test coatings in the concentration range 0.1–0.01 wt.% of metal in dry matter content of alkyd binder. Set-to-touch time (τ_1_), tack-free time (τ_2_), dry-hard time (τ_3_) and dry-through time (τ_4_), estimated by Beck–Koller method, are summarized for formulations of **S471** and **SP262** in Table 1 together with relative hardness determined by pendulum test 10 and 100 days after application. In the binder **S471**, the title compound is highly active in whole concentration range, as the dry-hard time does not exceeds 9 h. Typical feature of the binder is a fast film formation due to absence of low volatile solvents in the binder. Therefore, according to the Beck–Koller method, the coatings can be considered as set-to-touch dry in few minutes after application on test substrate even in the absence of a drier. It should be noted that similar behavior was observed for resin **S622**. Tack-free time of the formulations Mn/**S471** increases with decreasing concentration. However, similar dependency was not observed for parameters τ_2_, τ_3_, and τ_4_, which is probably a result of better through drying of formulations with the lower drier content. The main advantage of Mn is unusually high activity at a considerably lower concentration than recommended for cobalt(II) 2-ethylhexanoate (Co). Therefore, Mn shows satisfactory performance in **S471** at concentration 0.01 wt.%, whereas Co is almost inactive at the same level as evident from high values of τ_2_ (18.1 h). Furthermore, the coatings containing this commercial drier are not dry-through within 24 h. 

Additional experiments on commercial manganese compounds revealed that Mn provides shorter tack-free times than manganese 2-ethylhexanoate, but their overall performance is similar as their dry-hard times and try-through times are comparable. Low activity of manganese(II) acetate tetrahydrate, predissolved in DMSO, is attributed to presence of aqua ligands in the coordination sphere of manganese, which considerably prolongs initial phase of the autoxidation process (Table S1 in Supplementary Materials).

Promising drying activity of Mn was further evidenced in alkyd resin **SP262**. Therefore, dry-through time (τ_4_) shorter than 10 h is observed at the concentration range 0.1 to 0.03 wt.%. Commercial Co exhibits, at this concentration range, τ_3_ longer than 9 h and τ_4_ longer than 15 h.

Relative hardness of test coatings of Mn/**S471** and Mn/**SP262**, measured 10 days after application (*H*_rel;10d_), varies around 20%, which is ~5% points lower value than observed for coatings cured with Co. Final relative hardness of the coatings, treated with Mn, varies around 35%. The coatings, cured with Co, are considerably harder due to higher density of cross-linking, which is probably caused by a higher long-term stability of the catalytically active species. Note that the lower final hardness is a common feature of the alkyd coatings treated with cobalt alternatives [29]. Furthermore, the long-term stability of the active species, originating from cobalt carboxylates, has also some negative aspects, such as a tendency to early embrittlement and aging as the polymer degradation processes, which are also based on autoxidation and can be promoted by primary driers [30].

The results of mechanical tests, performed on formulation of **S622** and **SP252**, are summarized in Table 2. In both alkyd resins, manganese-based drier performs well at concentration range 0.1 to 0.01 wt.% giving shorter τ_3_ and τ_4_ than appropriate coatings treated with Co. Considerably better drying performance is evidenced mainly at low metal concentration where formulations Co**/S262** and Co**/SP252** give very long touch-free times (τ_2_). 

The cured films of alkyd resins **S622** and **SP252** show generally a lower final hardness than aforementioned resins **S471** and **SP262**. Such an effect occurs because of a different composition of the binder, due to a lower content of aromatic dibasic acid in the resins of long oil-length. Furthermore, the higher content of fatty acid chains results in a more effective film plasticization. The coatings cured with Mn show a lower final hardness then those treated with Co, which agrees with the trend observed for alkyds of medium oil length. It should be noted that dependence on the drier concentration is here more distinct due to a lower contribution of the phenylene building blocks on the polymer hardness. 

**Figure 1:**
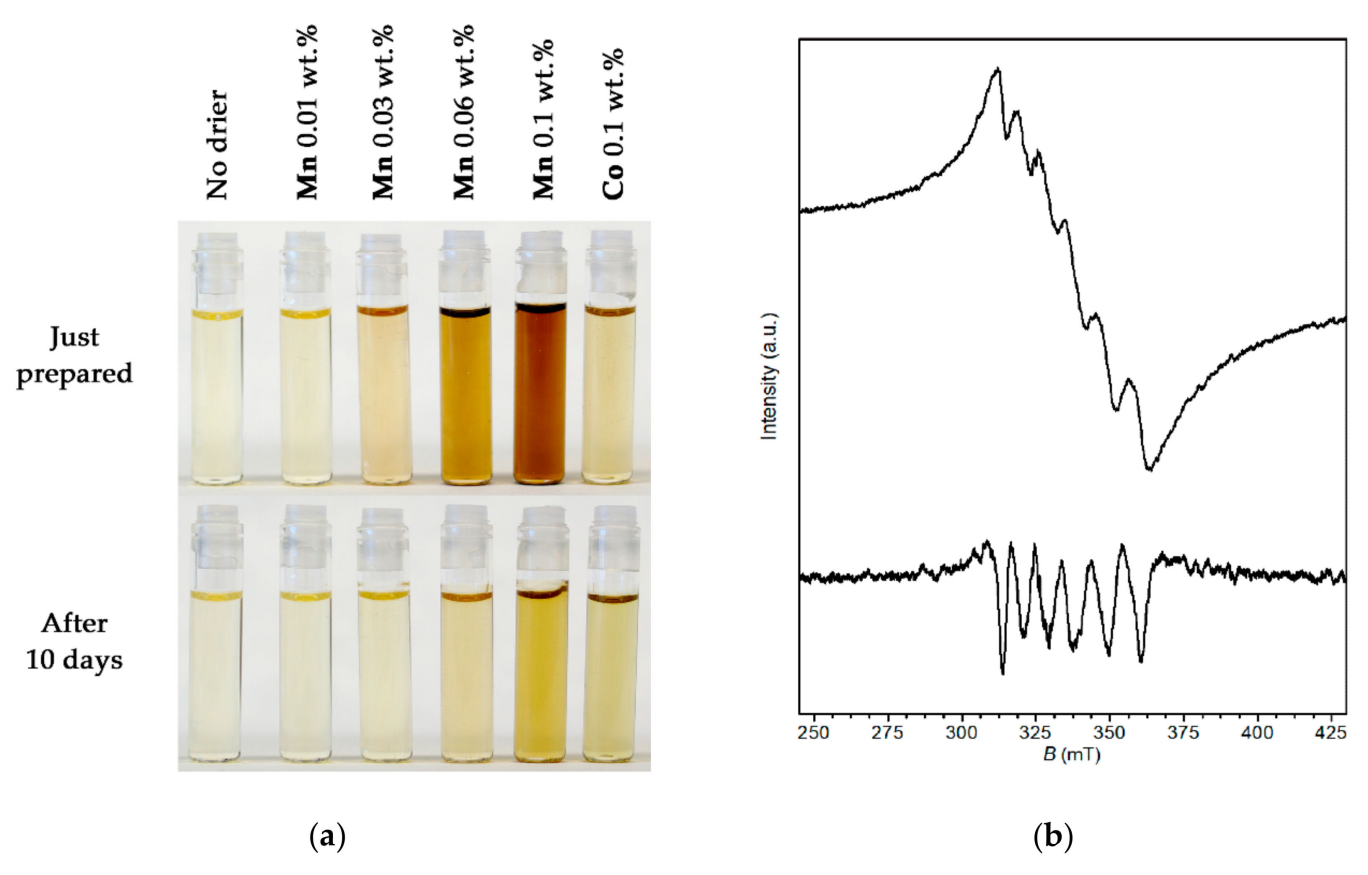
(**a**) Coloration of Mn**/S471** formulations and a comparison with samples of neat **S471** and Co**/S471** (0.1 wt.%); (**b**) Electron Paramagnetic Resonance (EPR) spectrum of Mn**/S471** formulation (0.1 wt.%; top) and its derivation (bottom) measured after 10 days of in closed vial (ν = 9.4274 GHz, |*A*_iso_| = 93.4 × 10^−4^ T, *g*_iso_ = 1.9985).

Application of manganese-based driers in paint formulations can be limited with their dark coloration, which affects manly colorless lacquers and light-pigmented enamels [31]. Although freshly prepared formulations of Mn are dark-brown colored at high concentrations, storage in closed vessels results in their discoloration in few days as documented on formulations of **S471** shown in Figure 1a. Such color change is due to reduction of Mn^III^ to Mn^II^, as proved by electron paramagnetic resonance (EPR). EPR spectrum of a sample measured after storage in a closed vessel for ten days exhibits a typical six-line pattern due to hyperfine interaction of unpaired electron with nucleus of ^55^Mn (*I* = 5/2), which natural abundance is 100% (Figure 1b). Calibration on freshly prepared formulation of manganese(II) acetate tetrahydrate in **S471** enabled to quantify amount of Mn^II^ in formulations of Mn/**S471**. Freshly prepared sample of metal concentration 0.1 wt.% contains about 12% of Mn^II^; 50% and 90% conversion of Mn^III^ to Mn^II^ was observed after one-day and seven-day storage, respectively. Our experiment with formulation of Mn/**S471** (0.03 wt.%), stored for one weak under inert atmosphere of nitrogen, revealed prolongation of tack-free time up to 2.7 h but overall performance seems to be similar to freshly prepared formulation as dry-hard time and dry-through time stay comparable.

Coloration of transparent paint films treated with Mn and Co was evaluated by spectroscopy in visible spectral region on formulations of **S471**. The measurements were done on coatings of 120-μm wet thickness in transmission mode in concentration range 0.1 to 0.01 wt.% of metal in dry matter content. We note that the coatings were cast from freshly prepared formulations and their color was evaluated 3 and 60 days after application. The colorimetric data, summarized in Table 3, revealed a strong chromatic shift to yellow (see parameter b*) only for formulation of Mn/**S471** at metal concentration 0.1 wt.%. In the range 0.06–0.03 wt.%, Mn shows acceptable values of b* as they near the values obtained for formulations of Co/**S471** at commonly used concentrations (0.1–0.06% wt.%). Negligible effect on color was observed at 0.01 wt.% of Mn. Its yellowish color is mainly due coloration of the alkyd binder itself. 

Note that the treatment of **S471** with Co also causes chromatic shift of the transparent coatings to greenish yellow, even though the drier solutions in inert solvents are purple. Surprisingly, the cobalt-based drier does not compensate yellowish appearance of the binder, as one could suggest, probably due to a redox process or chemical changes in the coordination sphere of cobalt. 

Only minor color changes were observed on samples of Mn**/S471** and Co**/S471** stored on a diffuse daylight for 60 days. They are attributed to yellowing of the alkyd binder upon storage on the diffuse daylight. We note that coloration of coatings (**S471**) treated with manganese(II) 2-ethylhexanoate is very similar to Mn**/S471** (Table S2 in Supplementary Materials). Only at concentration 0.1 wt.%, it shows lower yellowing index (b* = 0.70).

### 3.2. Performance in High-Solid Alkyd Binders

Promising activity of the title compound in solvent-borne alkyd formulations led us to investigate its drying performance in high-solid formulations. This type of binder is currently preferred due to a low content of volatile organic compounds, but is generally more sensitive to a proper choice of the primary drier owing to negligible effect of solvent evaporation on the film formation process. The tests were performed on alkyd resins **FP07** (solid content = 89%), **SP00** (solid content = 99.5%) and **TI870** (solid content = 98.9%). For our purposes, the latter two binders were diluted with dearomatized white spirit to 90% solid content in order to improve their film-forming properties. Drying times, estimated on test coatings of 38 and 76 µm wet thickness, are summarized in Table 4. Two sets of experiments were conducted since the high-solid binders are generally cured less homogenously due to limited diffusion of air-oxygen. The chemical curing of high-solid binders has to be faster than in the case of solvent-borne formulations in order to reach satisfactory drying times [32].

The binder **FP07** is well cured with Mn in the concentration range 0.1 to 0.01 wt.% as evident from dry-through times (τ_4_) of the 76 µm films those are used for standard tests of drying activity. Optimal performance is observed at 0.03 wt.% and only mild overdose effect is evidenced at higher concentrations. The 76 µm layers are cure almost homogenously as evident from comparison with 38-µm films. The cobalt based drier Co shows optimal performance 0.06 wt.%, where 76 µm coatings become through dry within 17.4 h. At higher or lower concentrations, the 76 µm coatings are not through dried within 24 h. Considerably lower values of τ_4_ were obtained for 38 µm films in concentration range 0.1–0.03 wt.% (7.1–9.9 h). This observation reveals a front formation and a limited oxygen penetration into the whole volume of the 76 µm alkyd layers.

Coatings of the binder **SP00** are well through-dried by Mn in the concentration 0.1–0.03 wt.% as the τ_4_ values of 76 µm films do not exceed 11 h. Cobalt based drier Co shows a pure through drying only at high concentrations (0.1–0.06 wt.%). Optimal dosage is observed at 0.03 wt.% where through drying is satisfactory and values of τ_1_–τ_3_ still very low. 

The coatings of **TI870** treated with Mn and Co cannot be considered as through-dried after 24 h of curing, as they stay soft for a long time. Therefore, dry-hard time (τ_3_) is used for comparison of a driers performance. The binder **TI870** shows a very strong tendency to front-formation, as evidenced on coatings treated with Co (cf., τ_3_ for 38 and 76 µm films at 0.1 and 0.06 wt.%). In case of Mn, smaller differences in τ_3_ of 38 and 76 µm films is observed. Nevertheless, at high concentrations (0.1–0.06 wt.%), they are considerably higher than in case of the other high-solid binders under the study. 

Relative hardness of the test coatings treated with Mn and Co are summarized in Table 5. As expected, the values are considerably lower than observed for solvent-borne systems, which is given by lower molecular weights of the fresh binders. Films of the high-solid binders, treated with Mn, show sufficient hardness already after 10 days of curing. The values of *H*_rel;10d_ is comparable with appropriate systems treated with Co (Table 5). Although the hardness development continues in both cases, the systems containing Co show higher final hardness (*H*_rel;100d_). The discrepancy is much stronger in case of the binder **TI870**, which is probably caused by different fatty acid pattern. **TI870** is produced from tall oil fatty acids while the other alkyd resins, under the study, are modified with vegetable oils or soybean oil.

### 3.3. Kinetics of Autoxidation Process in Alkyd Film

**Figure 2:**
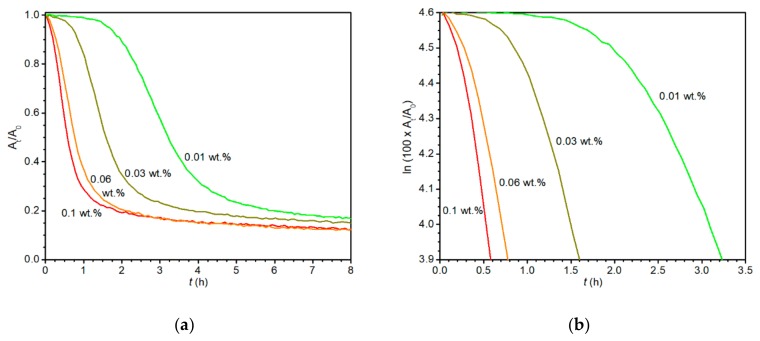
Time development of the infrared band ν_a_(*cis*-C=*C–H*) at 3008 cm^−1^ attributed to non-conjugated double bond system: (**a**) Linear plot for **S471**/Mn at different metal concentration; (**b**) logarithmic plot for **S471**/Mn.

**Figure 3:**
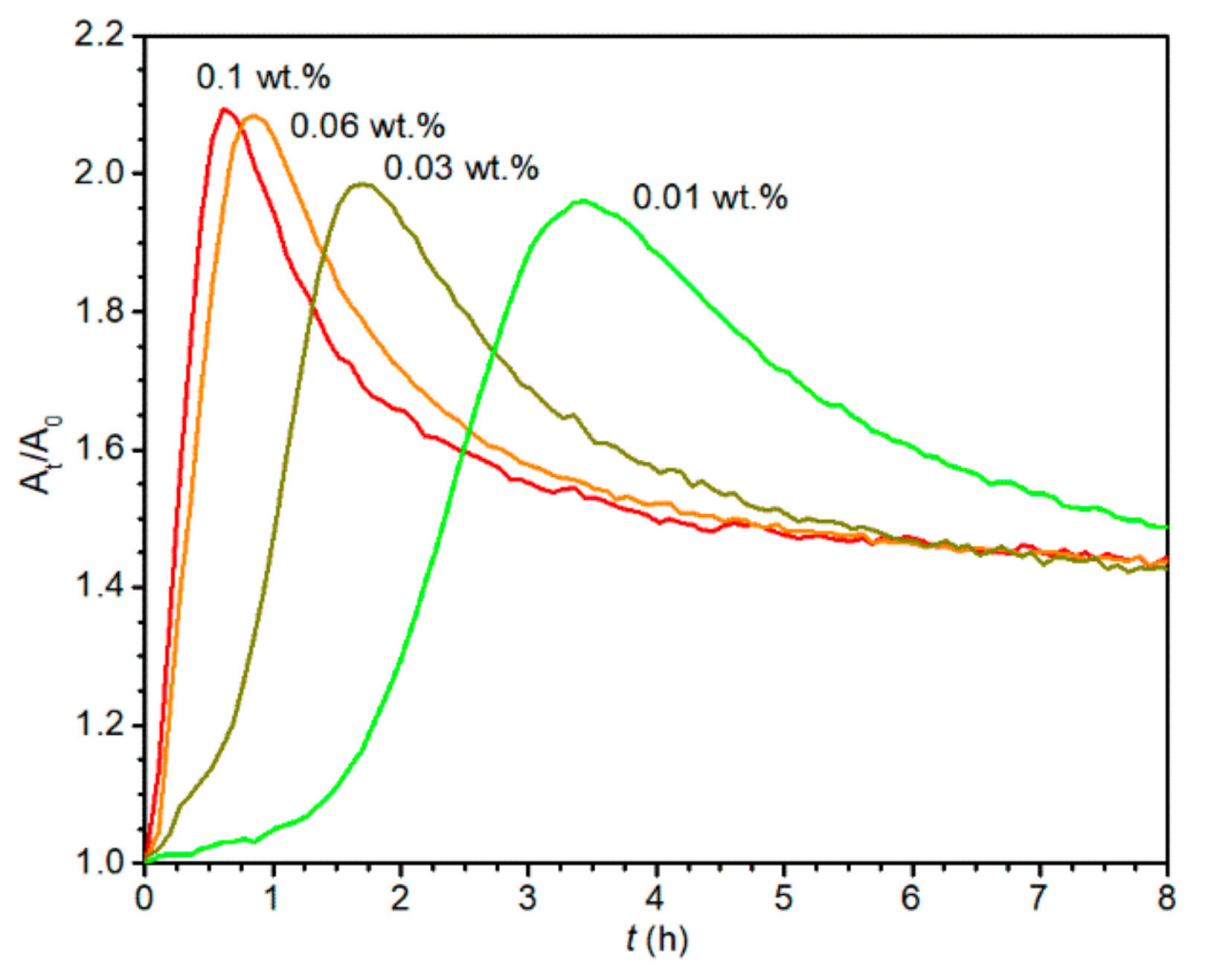
Time development of the infrared band ω(*cis*-*trans*-C=*C–H*) at 989 cm^−1^ attributed to conjugated double bond system.

The curing process in the alkyd resin **S471** was followed by time-resolved infrared spectroscopy. Such experimental method enables to follow autoxidation of unsaturated fatty acid chains of the alkyd resin through development of characteristic bands at 3008 and 989 cm^−1^ (Figure 2 and Figure 3). The band at 3008 cm^−1^ is assigned to stretching of C–H groups on the isolated double bonds in *cis*-configuration [ν_a_(*cis*-C=*C–H*)]. During the autoxidation process, the band decreases in intensity as peroxidation proceeds and isolated double bonds are converted to conjugated system of double bonds [1,6]. The appearance of conjugated double bonds can be followed on band at 989 cm^−1^ that is assigned to C–H wagging [ω(*cis-trans*-C=*C–H*)] [6]. 

The C–H stretching band at 3008 cm^−1^ is very suitable for the investigation of autoxidation kinetics as it follows consumption of the active substrate (Figure 2a). Based on our previous studies [6,30], the autoxidation of a thin layer of alkyd resins behaves as a reaction of pseudo-first-order until ~50% conversion, which is related with sufficient mobility of the system. In later phases of the process, the system becomes solid and the autoxidation slows down more rapidly than the pseudo-first order reactions as it no longer satisfy the rule of a stirred liquid, which becomes evident as deviation from linearity in the logarithmic plots. 

Our kinetic experiments were performed on thin films of the test formulations, which enable the elimination of the effect of air-oxygen diffusion. The estimated rate coefficients (*k*_max_), induction times (*t*_ind_) and reaction half-life (*t*_1/2_) for formulations **S471**/Mn are summarized in Table 6. In the concentration range 0.1–0.01 wt.%, the rate coefficient increases with increasing concentration, as evident from slopes of the time-development of [ν_a_(*cis*-C=*C–H*)] in the logarithmic scale, whereas induction time decreases (Figure 2b). The values of *k*_max_ are enough high and comparable with Co. High curing power at low concentration are apparently caused by short induction times. Therefore, at 0.01 wt.%, Mn shows still satisfactory value of *t*_ind_ (2.2 h) while initiation of the autoxidation by Co is tedious at this concentration (*t*_ind_ = 6.9 h). The estimated half-lives of the autoxidation well correlate with the drying times established by mechanical tests. Nevertheless, the overdose effect mentioned at 0.1 wt.% was not captured by kinetic measurements owing to the use of thinner alkyd layers. Such discrepancy supports the aforementioned explanation that the overdose is caused by thin polymeric film on the coating surface that restricts the oxygen diffusion into whole volume of the coating.

Development of the bands at 989 cm^−1^ evidences appearance of the conjugated double bond systems upon the autoxidation. The band reaches the maximum, at *t*_conj_, and then a decrease in intensity is observed (Figure 3). It is due to high reactivity of the conjugated double bonds and their participation in alkyd cross-linking [1]. The value of *t*_conj_ increases with decreased concentration and further documents a relatively fast process even at metal content 0.01 wt.%.

### 3.4. Thickness Effect

Effect of film thickness on rate of the autoxidation process was estimated by attenuated total reflectance infrared spectroscopy (ATR-IR). As recently demonstrated on high-solid formulations, the ATR sampling method enables to follow the autoxidation process on the interface coating/ATR crystal. Series of the measurements on samples of different film thickness is very suitable to approach curing process in the different parts of a thick alkyd layer and to distinguish inhomogeneity caused by limited air-oxygen diffusion [32].

Thickness effect was evaluated in formulation of Mn/**S471** at metal concentration 0.03 wt.%, which was chosen based on optimal performance in mechanical tests and aforementioned kinetic experiments. Only one formulation was chosen for the scrutiny, as the evaluation by ATR-IR method is highly time-consuming. 

**Figure 4:**
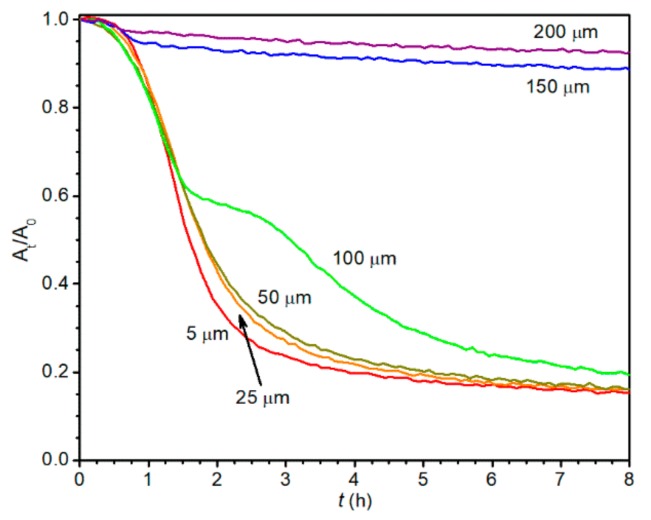
Development of ν_a_(*cis*-C=*C–H*) in time for coatings of Mn**/S471** (0.03 wt.%). Labels refer to wet thickness of given coatings.

Development of the characteristic band of ν_a_(*cis*-C=*C–H*) in time, measured for coatings of 5, 25, 50, 100, 150, and 200 µm wet thickness is given in Figure 4. The 25 and 50 µm coatings show a very similar development to the coating of 5 µm wet thickness, which was used for aforementioned kinetic measurements. It implies homogenous film drying and negligible effect of the air-oxygen diffusion in these films. In the case of 100 µm coating, a strong deceleration of the autoxidation is observed at ~40% conversion (*t* ≈ 1.5 h). Nevertheless, the process reaccelerates after next ~1.5 h, which is a typical feature of the front-forming drying [32]. Coatings of 150 and 200 µm wet thickness are not through dried within 24 h as evident from a negligible consumption of the isolated double bonds on the interface sample/ATR crystal. Such observation reveals a very slow movement of the front towards the bottom and its low permeability for the air-oxygen.

### 3.5. Raman Spectroscopy

The investigation of curing process in formulation Mn/**S471** by Raman spectroscopy was performed at concentration 0.03 wt.% on coatings of 60-µm wet thickness. Such arrangement ensures a homogenous curing of the alkyd layer with a negligible effect of oxygen diffusion as verified by aforementioned measurements of the thickness effects. 

**Figure 5:**
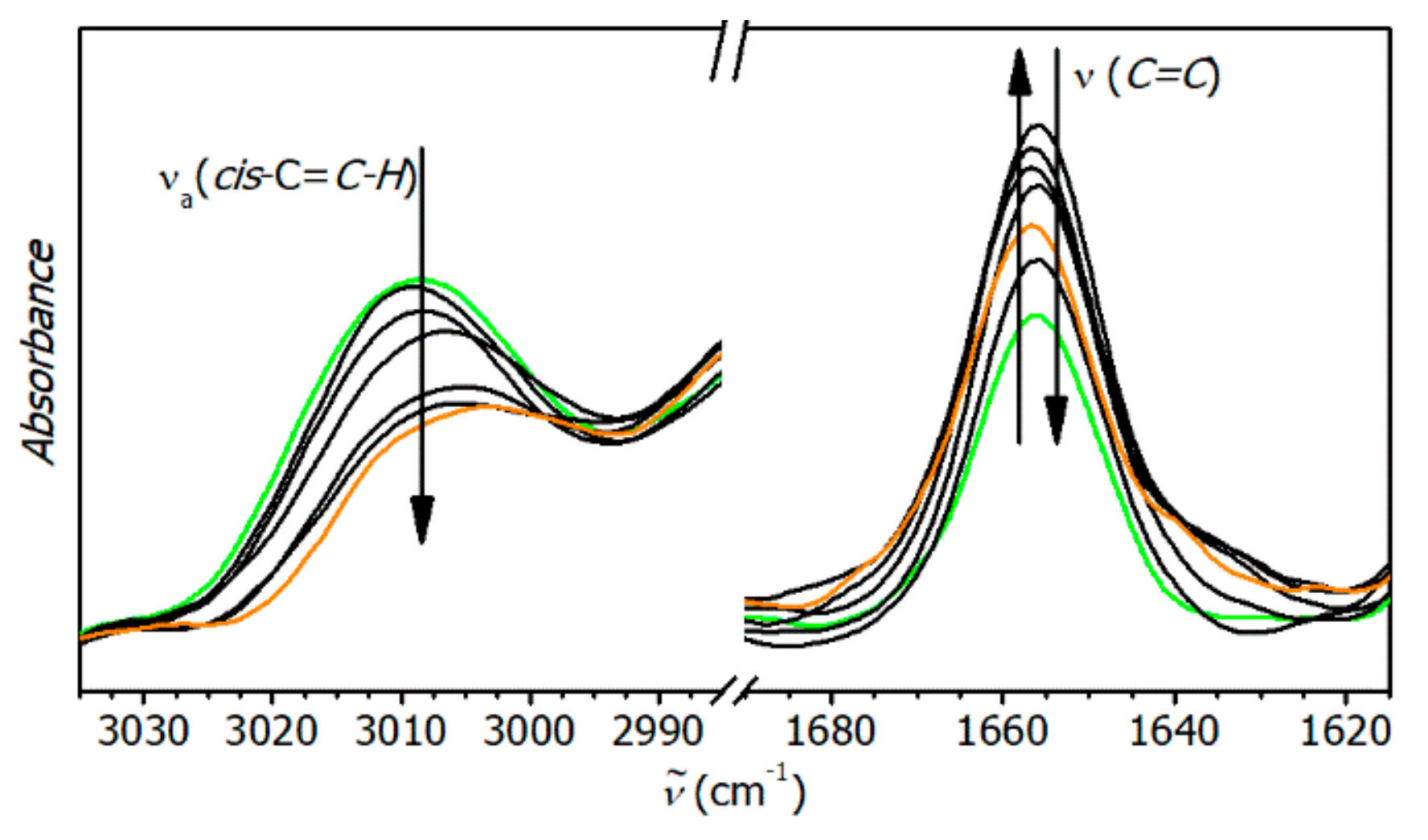
Development of the bands of ν_a_(*cis*-C=*C–H*) and ν(*C=C*) in the Raman spectra of Mn**/S471** (Green and orange lines represent the first and the last spectrum in the series, respectively.).

The collected Raman spectra reveal a strong development of the bands at 3008 and 1656 cm^−1^ in time (Figure 5), which is in agreement with previous studies on ethyl linoleate used as a liquid model system for alkyd resin [7]. The band at 3008 cm^−1^ has been assigned to C–H stretching of allylic group [ν_a_(*cis*-C=*C–H*)] of the unsaturated fatty acid chains [33]. It decreases in intensity due to conversion of the isolated double bonds to a conjugated system. Developments of the band at 1656 cm^−1^, assigned to stretching of aliphatic C=C bonds [33], also reflects the chemical changes in fatty acid chains upon the autoxidation. Initial increase in intensity is related to appearance of conjugated double bond system, which has higher extinction coefficient than isolated double bonds. Intensity of the band reaches maximum in ~2 h, which is in line with aforementioned data obtained from time-resolved infrared spectroscopy (see *t*_conj_ in Table 6). The following decrease in intensity is ascribed to extensive cross-linking, which is related with addition of alkoxyl and peroxyl radicals on double bonds and results in decrease of unsaturation degree [1].

## 4. Conclusions

In summary, the present study described a promising drying performance of Mn in various alkyd binders. The species, dissolved in dimethyl sulfoxide, shows an excellent performance at 0.03 wt.% in the used binders and, in particular cases, concentration 0.01 wt.% is satisfactory for common applications. The coatings, treated by Mn, are generally faster through-dried, when compared to those containing Co as evidenced by drying time measurements. Such behavior resembles other “cobalt alternatives” reported in literature [29,32]. Although Mn is dark colored in a pure form, storage of the formulations containing Mn as well as transparent film treated with Mn exhibit acceptable coloration at metal concentration 0.03 wt.% due to Mn^III^ to Mn^II^ reduction as evidenced by EPR spectroscopy. On formulation Mn/**S471**, kinetics of the autoxidation process was followed by time resolved spectroscopic experiments. The measurements revealed similar rate coefficients (*k*_max_) to Co/**S471** at the same concentration and considerably better performance at low metal concentrations is apparently due to shorter induction times (*t*_ind_). Through drying of the formulation Mn/**S471** at 0.03 wt.% was investigated by IR/ATR technique. It proves homogenous drying up to ~100-μm wet thickness without use of other additives. 

## Figures and Tables

**Table 1:** Drying times and relative hardness for formulations of medium oil-length alkyd resins.

Binder	Drier	C (wt.%)	τ_1_ (h)	τ_2_ (h)	τ_3_ (h)	τ_4_ (h)	*H*_rel;10d_ (%)	*H*_rel;100d_ (%)
**S471**	Mn	0.1	-	0.5	7.3	9.9	19.5	35.8
		0.06	-	0.8	9.0	12.2	19.6	35.2
		0.03	-	2.0	7.0	10.3	20.0	35.1
		0.01	-	3.5	8.3	12.3	19.1	33.4
	Co	0.1	-	0.4	7.7	>24	22.5	49.1
		0.06	-	1.1	7.1	>24	29.7	49.7
		0.03	-	1.2	9.3	>24	24.5	46.2
		0.01	-	18.1	>24	>24	24.5	43.6
**SP262**	Mn	0.1	0.8	1.2	7.6	9.3	18.2	35.3
		0.06	1.2	1.8	7.6	7.9	20.2	34.0
		0.03	1.6	2.3	7.2	7.9	20.0	35.2
		0.01	5.6	6.5	10.0	12.0	19.8	33.6
	Co	0.1	0.4	1.0	9.7	>24	25.5	49.1
		0.06	0.9	1.7	13.2	15.9	23.1	47.2
		0.03	2.5	3.3	10.8	17.3	25.0	39.4
		0.01	11.9	14.0	16.6	20.2	24.0	36.2

**Table 2:** Drying times and relative hardness for formulations of long oil-length alkyd resins.

Binder	Drier	C (wt.%)	τ_1_ (h)	τ_2_ (h)	τ_3_ (h)	τ_4_ (h)	*H*_rel;10d_ (%)	*H*_rel;100d_ (%)
**S622**	Mn	0.1	-	0.6	4.0	6.3	17.7	20.4
		0.06	-	0.8	4.4	7.5	16.4	18.6
		0.03	-	1.8	5.5	9.1	16.9	18.0
		0.01	-	3.2	7.6	9.8	15.3	16.6
	Co	0.1	-	0.9	4.1	12.8	21.9	43.4
		0.06	-	1.9	6.4	11.8	19.3	39.7
		0.03	-	3.7	5.9	10.5	17.7	37.0
		0.01	-	11.2	14.4	17.8	14.7	30.9
**SP252**	Mn	0.1	1.0	1.6	6.9	9.2	11.6	20.6
		0.06	1.3	2.2	7.5	9.3	11.0	20.4
		0.03	2.2	2.8	7.9	10.4	11.1	19.9
		0.01	2.7	4.3	7.4	10.8	10.7	19.2
	Co	0.1	0.4	0.5	8.7	18.4	15.2	35.1
		0.06	0.9	1.1	15.8	18.9	13.7	32.5
		0.03	3.7	4.5	11.1	16.9	13.1	30.2
		0.01	14.3	14.8	15.7	19.9	– ^1^	– ^1^

^1^ Not measured.

**Table 3:** Coloration of coatings of **S471** treated with Mn and Co. ^1^

Binder	Drier	C (wt.%)	*L	*a	*b
**S471**	Mn	0.1	99.4/99.7	−0.10/–0.17	0.95/0.92
		0.06	99.6/99.8	−0.07/–0.12	0.47/0.43
		0.03	99.6/99.6	−0.07/–0.10	0.33/0.31
		0.01	99.9/99.8	–0.06/–0.10	0.16/0.26
	Co	0.1	99.7/99.6	−0.17/–0.12	0.50/0.63
		0.06	99.7/99.8	−0.15/–0.13	0.42/0.45
		0.03	99.4/99.9	−0.10/–0.08	0.26/0.31
		0.01	99.6/99.8	–0.06/–0.09	0.14/0.28

^1^ Values “*m*/*n*” signify data collected 3 days (*m*) and 60 days after application (*n*). Wet thickness: 120-μm.

**Table 4:** Drying times of test coatings for formulations of high-solid alkyd binders.^1.^

Binder.	Drier	C (wt.%)	τ_1_ (h)	τ_2_ (h)	τ_3_ (h)	τ_4_ (h)
**FP07**	Mn	0.1	1.6/1.7	2.7/5.2	6.6/8.8	6.6/8.8
		0.06	2.0/2.2	3.1/5.7	6.1/8.5	6.1/8.5
		0.03	3.3/3.4	4.4/4.6	5.5/7.4	6.7/7.4
		0.01	5.0/5.2	6.2/6.6	7.5/7.5	7.5/7.7
	Co	0.1	0.9/1.1	2.2/2.8	5.5/10.4	9.9/>24
		0.06	1.1/1.2	2.5/3.2	4.3/11.3	7.1/17.4
		0.03	1.8/2.2	2.6/6.7	5.9/11.3	7.9/>24
		0.01	6.3/6.7	7.8/7.9	8.6/9.4	15.0/>24
**SP00**	Mn	0.1	1.4/1.5	1.8/2.4	2.7/3.0	4.1/8.6
		0.06	1.5/2.0	2.4/3.3	2.8/3.7	7.1/9.2
		0.03	2.8/3.1	3.2/3.5	4.5/5.1	7.0/10.1
		0.01	5.9/6.1	6.3/6.7	8.8/8.8	11.8/12.4
	Co	0.1	0.2/0.2	0.3/1.1	0.7/2.7	>24/>24
		0.06	0.7/0.5	1.3/1.3	1.3/2.0	13.2/>24
		0.03	1.2/0.9	2.1/1.7	2.1/4.5	3.0/10.7
		0.01	3.3/2.9	5.7/4.9	5.7/4.9	9.8/10.6
**TI870**	Mn	0.1	2.0/2.1	2.7/3.6	3.6/8.8	18.8/>24
		0.06	2.6/2.5	3.5/4.1	3.8/8.1	>24/>24
		0.03	4.1/3.8	5.2/4.9	5.2/8.0	>24/>24
		0.01	9.0/8.5	10.3/10.0	11.8/11.2	>24/>24
	Co	0.1	0.4/0.6	2.3/8.0	8.1/>24	8.1/>24
		0.06	1.2/1.3	1.6/4.0	8.8/>24	>24/>24
		0.03	2.2/2.2	2.8/6.1	5.0/9.0	>24/>24
		0.01	4.4/4.6	5.4/5.7	6.3/11.3	>24/>24

^1^ Values “*m*/*n*” signify drying time for coating of 38 µm wet thickness (*m*) and 76 µm wet thickness (*n*).

**Table 5:** Relative hardness (in %) of test coatings for formulations of high-solid alkyd binders.^1.^

Drier	C (wt.%)	FP07	SP00	TI870
Mn	0.1	7.9/11.7	10.4/11.3	6.5/13.2
	0.06	7.4/10.7	10.0/11.1	6.6/13.4
	0.03	6.6/9.6	9.8/11.2	6.6/10.6
	0.01	6.1/8.0	9.7/10.7	5.5/9.4
Co	0.1	8.7/16.6	9.0/17.2	9.7/27.2
	0.06	8.0/13.8	8.4/15.6	8.8/24.3
	0.03	7.3/10.6	8.3/12.8	7.3/19.8
	0.01	6.5/10.8	8.5/11.0	6.3/14.8

^1^ Values “*m*/*n*” signify relative hardness estimated 10 days (*m*) and 100 days after application (*n*).

**Table 6:** Kinetic parameters for Mn/**S471** and Co/**S471**.^1.^

Binder	Drier	C (wt.%)	*t*_ind_ (h)	*k*_max_ (h^−1^)	*t*_1/2_ (h)	*t*_Conj_ (h)
**S471**	Mn	0.1	0.2	1.85	0.6	0.6
		0.06	0.3	1.46	0.8	0.9
		0.03	0.9	1.05	1.6	1.7
		0.01	2.2	0.65	3.2	3.4
	Co	0.1	0.2	2.18	0.5	0.6
		0.06	0.5	1.55	0.9	1.0
		0.03	2.0	0.81	2.9	3.0
		0.01	6.9	0.45	8.4	8.7

^1^ Data estimated by time-resolved infrared spectroscopy using ATR sampling method.

## Supplementary Materials

The following are available online at https://www.mdpi.com/1996-1944/13/3/642/s1, Table S1: Drying times for formulations of **S471** treated with various manganese compounds; Table S2: Coloration of coatings of **S471** treated with manganese 2-ethylhexanoate.
