# Supplementary material for: Performance of Manganese(III) Acetylacetonate in Solvent-Borne and High-Solid Alkyd Formulations
Source: Materials (Basel). 2020 Feb 1;13(3):642. doi: 10.3390/ma13030642 (PMC7041375; doi:10.3390/ma13030642)
Supplement: Supplementary file 1 [file materials-13-00642-s001.pdf]

materials

Supplementary Materials

Performance of Manganese(III) Acetylacetonate in Solvent-Borne and High-Solid Alkyd Formulations

Eliška Matušková and Jan Honzíček *

Institute of Chemistry and Technology of Macromolecular Materials, Faculty of Chemical Technology, University of Pardubice, Studentská 573, 532 10 Pardubice, Czech Republic; st42098@student.upce.cz
* Correspondence: jan.honzicek@upce.cz; Tel.: +420-466-037-229

Table S1. Drying times for formulations of **S471** treated with various manganese compounds.[1]

Binder	Drier	C (%)	τ_1 (h)	τ_2 (h)	τ_3 (h)	τ_4 (h)
**S471**	**Mn(acac)$_3$**	0.1	-	0.3	4.3	10.7
		0.06	-	0.8	5.5	9.2
		0.03	-	1.8	6.8	9.2
		0.01	-	3.4	5.2	8.9
	**Manganese 2-ethylhexanoate**	0.1	-	2.1	5.8	13.8
		0.06	-	2.3	6.5	13.8
		0.03	-	3.3	5.7	12.5
		0.01	-	6.1	7.9	13.5
	**Manganese(II) acetate tetrahydrate**	0.1	-	4.8	9.3	14.7
		0.06	-	5.5	8.7	12.2
		0.03	-	7.7	8.5	12.0
		0.01	-	10.0	11.3	12.7

[1] Different alkyd bath was used than in the case of experiments reported in the main text.

Table S2. Coloration of coatings of **S471** treated with manganese 2-ethylhexanoate.[1]

C (%)	*L	*a	*b
0.1	99.8	–0.04	0.70
0.06	99.8	–0.05	0.50
0.03	99.8	–0.04	0.31
0.01	99.6	–0.05	0.18

[1] Data collected 3 days after application. Wet thickness: 120-µm.
